# Substance Use–Associated Infective Endocarditis Mortality in Young U.S. Adults, 2018 to 2024

**DOI:** 10.1016/j.jacadv.2026.103181

**Published:** 2026-09-23

**Authors:** Mafaz Mansoor, Janita Zarrish, Patrick Joseph, Rawa Salih, Matthew Varn, Matthew Brown, Daryoosh Derakhshan

**Affiliations:** a Department of Internal Medicine, South Georgia Medical Center, Valdosta, Georgia, USA; b Mercer University School of Medicine, Macon, Georgia, USA; c Department of Population Health, London School of Hygiene & Tropical Medicine, London, United Kingdom; d Division of Infectious Disease, South Georgia Medical Center, Valdosta, Georgia, USA; e Division of Cardiology, South Georgia Medical Center, Valdosta, Georgia, USA

**Keywords:** health disparities, infective endocarditis, mortality, opioid use, substance use, young adults

## Abstract

**Background:**

Substance use is a major driver of infective endocarditis (IE) in young U.S. adults, but recent national mortality trends remain unclear.

**Objectives:**

The objective of the study was to characterize trends in substance use-associated IE (SU-IE) mortality among U.S. adults aged 25 to 44 years, with expanded and sensitivity analyses.

**Methods:**

We analyzed Centers for Disease Control and Prevention Wide-ranging Online Data for Epidemiologic Research Multiple Cause of Death data from 2018 to 2024. IE-associated deaths were identified using International Classification of Diseases-10th Revision codes I33.0, I33.9, and I38; SU-IE required concurrent F11, F12, F13, F14, F15, or F19 codes. Mortality rates, overlap proportions, subgroup trends, and joinpoint annual percent changes with 95% CIs were assessed.

**Results:**

Among 9,745 IE-associated deaths in adults aged 25 to 44 years, 3,756/9,745 (38.5%) had substance-use overlap. Annual SU-IE deaths peaked at 655/1,604 (40.8%) in 2020 and 655/1,643 (39.9%) in 2021, then declined to 338/1,051 (32.2%) in 2024. The age-adjusted rate decreased from 0.75 per 100,000 (95% CI: 0.69-0.80) in 2021 to 0.41 (95% CI: 0.37-0.45) in 2024. Overlap decreased from 604/1,447 (41.7%) in 2018 to 338/1,051 (32.2%) in 2024. Significant post-2021 declines occurred among females (annual percent change, −23.59%), adults aged 25 to 34 years (−31.73%), adults aged 35 to 44 years (−12.55%), and White adults (−23.46%). American Indian or Alaska Native adults had the highest rates, and nonurban counties exceeded large central metropolitan counties. Among adults aged ≥25 years, 5,967/115,096 (5.2%) IE-associated deaths met the SU-IE criteria, including 2,211/5,967 (37.1%) among those aged ≥45 years.

**Conclusions:**

SU-IE mortality in young U.S. adults declined after 2021 but remained marked by demographic and geographic disparities.

Infective endocarditis (IE) is a serious cardiovascular and infectious disease associated with significant morbidity and premature mortality.^1^ Although structural heart disease, prosthetic valves, hemodialysis, and health care exposure have long been recognized as major risk factors for IE, the burden of disease in younger adults is now increasingly driven by substance use and infections related to injection practices.^2^^,^^3^ Previous national studies have shown a concerning increase in IE-related mortality among younger adults. A recent analysis found a concerning rise in substance abuse–associated IE mortality in young adults, with substance abuse documented in approximately one-third of deaths.^4^

However, trends in the most contemporary postpandemic era, particularly from 2021 through 2024, remain poorly characterized. During and following the COVID-19 pandemic, changes may have occurred in substance-use behaviors, health care access, harm reduction resources, overdose mortality patterns, and death certificate coding practices. Centers for Disease Control and Prevention (CDC) Wide-ranging Online Data for Epidemiologic Research (WONDER) Multiple Cause of Death data offer national, population-based mortality information derived from U.S. death certificates, including 1 underlying cause of death and up to 20 contributing causes, thereby allowing assessment of IE-associated mortality and concurrent substance use coding.^5^

We therefore performed a serial cross-sectional analysis of IE-associated mortality among U.S. adults aged 25 to 44 years using CDC WONDER data from 2018 through 2024. We evaluated temporal trends in all IE and substance use–associated IE (SU-IE) mortality, demographic, urbanization, and geographic disparities, and subgroup-specific changes using Joinpoint regression. We also performed expanded adult and sensitivity analyses to assess the age distribution and robustness of the findings.

## Methods

### Data source

This study utilized the Single Race data set from the CDC WONDER Multiple Cause of Death database, encompassing the years 2018 through 2024. The database contains annual national mortality and population data for U.S. residents derived from death certificates submitted to the National Vital Statistics System. Each certificate includes a single underlying cause of death, up to 20 contributing causes, and demographic information including sex, age, race, Hispanic origin, state of residence, and urbanization category.^5^ Because the data are publicly accessible and contain no individually identifiable information, this analysis did not constitute human subjects research and did not require institutional review board approval.

### Study population

The study population included U.S. decedents aged 25 to 44 years from 2018 through 2024. This age group was selected because prior national studies have demonstrated disproportionate IE mortality acceleration and substance use overlap among young adults.^4^^,^^6^ To contextualize findings across the broader adult population, we also performed an expanded age-stratified analysis including all decedents aged ≥25 years. County urbanization was classified using the 2023 National Center for Health Statistics (NCHS) Urban-Rural Classification Scheme, which assigns each county to 1 of 6 categories based on Office of Management and Budget metropolitan and micropolitan statistical area designations together with county population size: large central and large fringe metropolitan counties (metropolitan areas of ≥1 million population, subdivided by principal-city population containment), medium and small metropolitan counties (metropolitan areas of 250,000-999,999 and <250,000 population, respectively), and micropolitan and noncore nonmetropolitan counties.^7^ Micropolitan and noncore counties were considered the least urbanized county categories for descriptive analyses.

### Case definitions

IE-associated deaths were identified using multiple-cause-of-death International Classification of Diseases-10th Revision (ICD-10) codes: I33.0 (acute and subacute IE), I33.9 (acute endocarditis, unspecified), and I38 (endocarditis, valve unspecified). These codes were selected based on prior validated methodology for national IE mortality analyses.^4^^,^^6^^,^^8^

SU-IE deaths were defined as IE-associated deaths with concurrent substance use ICD-10 codes: F11 (opioid-related disorders), F12 (cannabinoid-related disorders), F13 (sedative-, hypnotic-, or anxiolytic-related disorders), F14 (cocaine-related disorders), F15 (other stimulant–related disorders), and F19 (multiple drug use or other psychoactive substance–related disorders). F16, F17, and F18 were excluded: F17 (nicotine dependence) is not directly linked to IE pathogenesis via injection; F16 (hallucinogen-related disorders) and F18 (volatile solvent-related disorders) were excluded because of limited IE relevance and low expected counts.

### Outcomes

The primary outcomes were annual SU-IE mortality counts and crude mortality rates per 100,000 among adults aged 25 to 44 years. Secondary outcomes included all IE mortality counts, age-adjusted IE and SU-IE mortality rates, and the substance-use overlap proportion, defined as: substance use overlap = (SU-IE deaths/all IE deaths) × 100.

Additional secondary outcomes included SU-IE mortality counts and age-adjusted rates among adults aged ≥25 years. Sensitivity analyses repeated the primary adult analysis after excluding cannabinoid-related codes (F12) and separately restricted the definition to opioid-related codes (F11).

### Stratification variables

Mortality was stratified by calendar year, sex, age group, race using CDC WONDER Single Race 6 categories, 2023 county urbanization category, and state of residence. Primary age–stratified analyses compared adults aged 25 to 34 and 35 to 44 years; expanded adult analyses used 10-year age groups among adults aged ≥25 years. Hispanic origin was evaluated in exploratory analyses but was not included in the primary race-stratified analysis because CDC WONDER captures Hispanic origin as a separate ethnicity variable rather than a race category.

### Statistical analysis

Deaths, crude and age-adjusted mortality rates, 95% CIs, and overlap proportions were summarized using CDC WONDER output. Annual mortality rates were calculated using the corresponding CDC WONDER Single Race population estimates for each calendar year and age-defined population as denominators. These denominator definitions were applied consistently across the primary (25-44 years) and expanded (≥25 years) analyses. Temporal trends were visualized using line graphs. Joinpoint regression (National Cancer Institute Joinpoint Regression Program, version 6.0.1) was performed using a grid search algorithm, allowing a minimum of 0 and a maximum of 1 joinpoint, with at least 2 observations between adjacent joinpoints and between a joinpoint and the end of the data series. For each evaluated mortality-rate series, the average annual percent change (APC) (AAPC) and corresponding 95% CIs were estimated for the full 2018 to 2024 study period. The optimal model was selected using the Monte Carlo permutation test (4,499 permutations; overall α = 0.05). Variance was modeled using the known SEs provided by the CDC WONDER reference data sets, assuming uncorrelated errors. Statistical significance was defined as 2-sided *P* < 0.05; significant APCs are denoted by an asterisk in the Joinpoint figures. Urbanization-specific population denominators were available through 2021; therefore, rate-based urbanization comparisons were limited to 2018 through 2021, whereas 2022 through 2024 analyses used death counts only. State-level analyses used combined 2018 through 2024 data to reduce suppression. Geographic comparisons were based on age-adjusted state-specific mortality rates standardized to the 2000 U.S. standard population. Suppressed cells and unstable estimates were handled according to CDC WONDER reporting rules.

Crude and age-adjusted mortality rate ratios (RRs) were calculated to compare mortality across demographic and geographic subgroups using pooled mortality rates over the study period. RRs were estimated by dividing the mortality rate in each comparison group by that of a prespecified reference group. Ninety-five percent CIs were calculated on the logarithmic scale using the SEs of the CDC WONDER mortality rates and the delta method. Age-adjusted RRs were calculated for sex and race comparisons. Crude RRs were calculated for comparisons between fixed age strata and urbanization categories because age-adjusted rates were not applicable or unavailable for these analyses.

## Results

### National trends

From 2018 through 2024, there were 9,745 IE-associated deaths among U.S. adults aged 25 to 44 years. Annual all-IE deaths increased from 1,447 in 2018 to a peak of 1,643 in 2021, then declined to 1,449 in 2022, 1,169 in 2023, and 1,051 in 2024. The age-adjusted all-IE mortality rate increased from 1.66 per 100,000 in 2018 to 1.87 per 100,000 in 2020 to 2021, then declined to 1.19 per 100,000 in 2024 (Table 1).

**Table 1:** National Trends in Infective Endocarditis and Substance Use-Associated Infective Endocarditis Mortality Among U.S. Adults Aged 25 to 44 Years, 2018-2024

Year	All IE Deaths	All IE Age-Adjusted Rate (95% CI)	SU-IE Deaths	SU-IE Age-Adjusted Rate (95% CI)	SU Overlap (%)
2018	1,447	1.66 (1.58-1.75)	604	0.70 (0.65-0.76)	41.7
2019	1,382	1.62 (1.53-1.71)	604	0.70 (0.65-0.76)	43.7
2020	1,604	1.87 (1.78-1.97)	655	0.75 (0.69-0.80)	40.8
2021	1,643	1.87 (1.78-1.97)	655	0.75 (0.69-0.80)	39.9
2022	1,449	1.68 (1.60-1.77)	529	0.60 (0.55-0.65)	36.5
2023	1,169	1.35 (1.27-1.43)	371	0.41 (0.37-0.45)	31.7
2024	1,051	1.19 (1.12-1.27)	338	0.41 (0.37-0.45)	32.2
Total	9,745	1.63 (1.60-1.66)	3,756	0.60 (0.58-0.62)	38.5

Mortality rates are expressed per 100,000 population and were age-standardized to the 2000 U.S. standard population. The substance use overlap represents the proportion of all infective endocarditis deaths in which at least 1 substance use-related diagnosis code was also recorded and was calculated as SU-IE deaths divided by all IE deaths. Values in parentheses represent 95% CIs. The total row represents the pooled 2018 to 2024 period estimate.

IE = infective endocarditis; SU = substance use; SU-IE = substance use-associated infective endocarditis.

**Figure 1:**
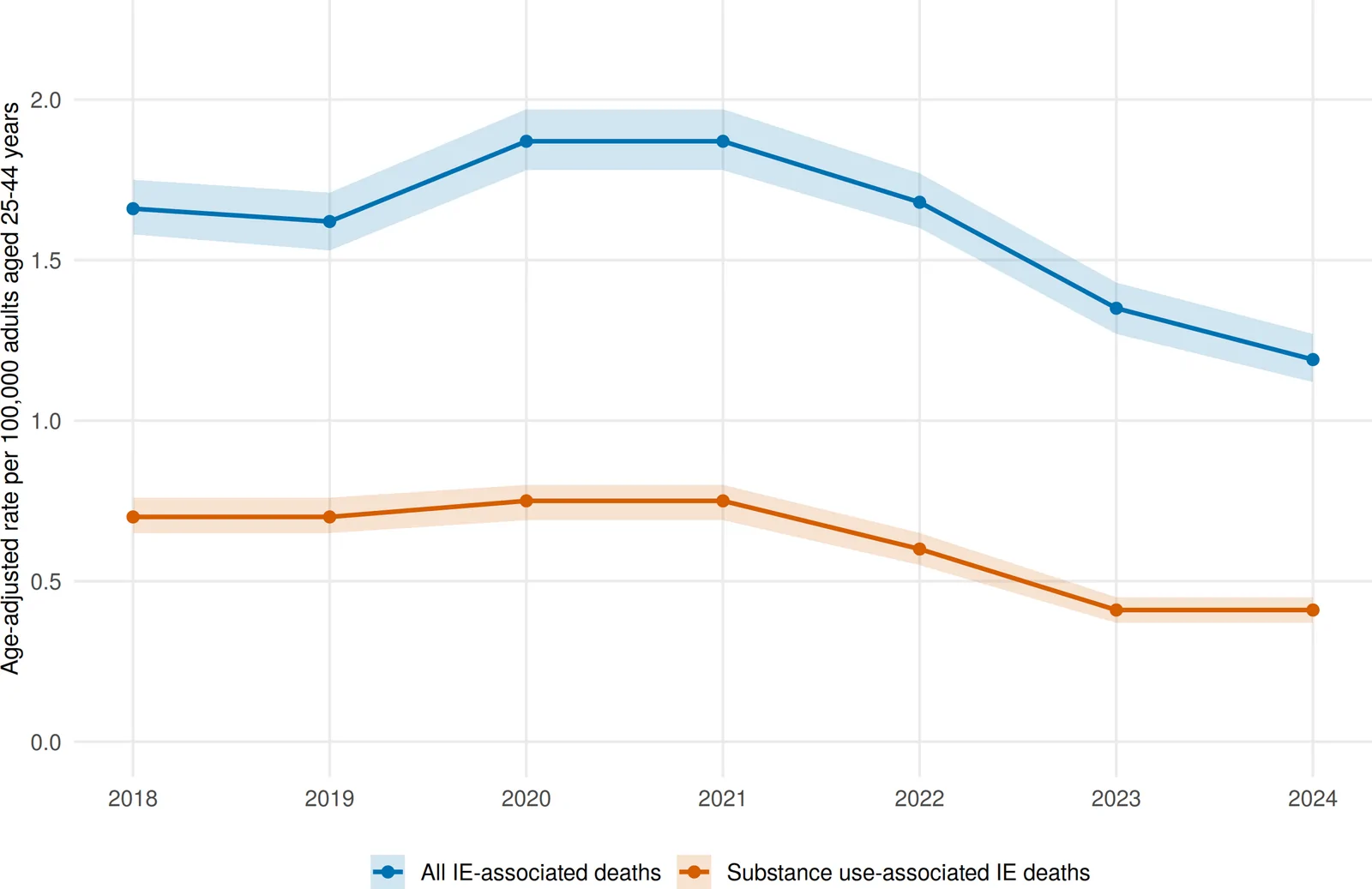
Trends in Infective Endocarditis and Substance Use–Associated Infective Endocarditis Mortality Among U.S. Adults Aged 25 to 44 Years, 2018 to 2024 Annual age-adjusted mortality rates per 100,000 population are shown for all infective endocarditis deaths and substance use–associated infective endocarditis deaths. Points represent annual mortality rates, lines represent temporal trends, and shaded bands indicate 95% CIs. IE = infective endocarditis.

SU-IE deaths totaled 3,756/9,745 (38.5%) over the study period. Annual SU-IE deaths were stable at 604/1,447 (41.7%) in both 2018 and 604/1,382 (43.7%) in 2019, increased to 655/1,604 (40.8%) deaths in 2020 and 655/1,643 (39.9%) in 2021, and declined thereafter to 529/1,449 (36.5%) in 2022, 371/1,169 (31.7%) in 2023, and 338/1,051 (32.2%) in 2024. The SU-IE age-adjusted mortality rate increased from 0.70 per 100,000 (95% CI: 0.65-0.76) in 2018 to 0.75 per 100,000 (95% CI: 0.69-0.80) in 2020 and 2021, then declined to 0.41 per 100,000 (95% CI: 0.37-0.45) in 2023 and 2024. Joinpoint regression showed no significant change from 2018 to 2021 (APC: 2.01%; 95% CI: −23.59% to 36.18%), followed by a nonsignificant decline from 2021 to 2024 (APC: −20.33%; 95% CI: −43.55% to 12.44%); the overall AAPC was −9.85% (95% CI: −18.62% to −0.13%; *P* = 0.047) (Supplemental Table 7). The proportion of IE deaths with substance-use overlap decreased from 604/1,447 (41.7%) in 2018 to 338/1,051 (32.2%) in 2024, with the steepest decline occurring after 2021 (Figure 1). In the expanded adult analysis including decedents aged ≥25 years, there were 115,096 IE-associated deaths from 2018 through 2024. The overall age-adjusted mortality rate was 6.34 per 100,000 (95% CI: 6.31-6.38) for all IE. Of these, 5,967/115,096 (5.2%) met the criteria for SU-IE, corresponding to an overall age-adjusted mortality rate of 0.41 per 100,000 (95% CI: 0.40-0.42). Annual adult SU-IE deaths increased from 891 in 2018 to 1,024 in 2021 and then declined to 642 in 2024. The age-adjusted adult SU-IE mortality rate declined from 0.50 per 100,000 (95% CI: 0.47-0.53) in 2021 to 0.30 per 100,000 (95% CI: 0.28-0.33) in 2024.

AAPCs with corresponding 95% CIs for all Joinpoint analyses are presented in Supplemental Table 7. Crude and age-adjusted mortality RRs for subgroup comparisons are presented in Supplemental Table 8.

### Sex-stratified trends

All-IE mortality was higher among males throughout the study period. Total male deaths were 5,355/9,745 (55.0%) compared with 4,390/9,745 (45.0%) female deaths. In 2024, males had 637/1,051 (60.6%) all-IE deaths (age-adjusted mortality rate, 1.45 per 100,000) compared with 414/1,051 (39.4%) female deaths (0.93 per 100,000).

**Figure 2:**
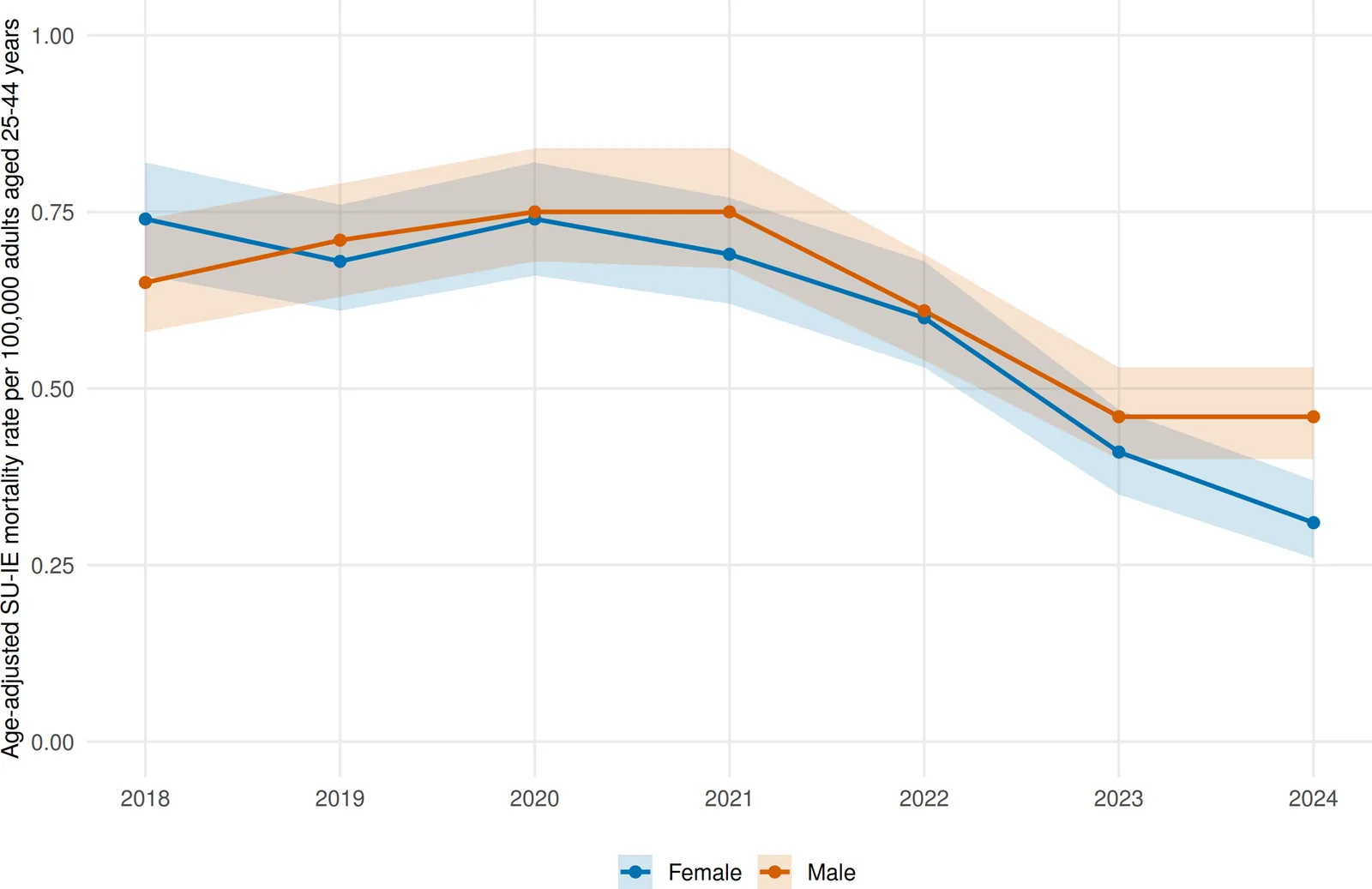
Sex-Specific Trends in Substance Use–Associated Infective Endocarditis Mortality Among U.S. Adults Aged 25 to 44 Years, 2018 to 2024 Annual age-adjusted mortality rates per 100,000 population are shown for males and females. Points represent annual mortality rates, lines represent temporal trends, and shaded bands indicate 95% CIs. SU-IE = substance use–associated infective endocarditis.

Conversely, females had consistently higher proportional substance use overlap. In 2018, SU-IE overlap was 328/701 (46.8%) among females compared with 276/746 (37.0%) among males. By 2024, overlap was 139/414 (33.6%) among females and 199/637 (31.2%) among males. Sex-stratified SU-IE mortality rates were relatively stable from 2018 to 2021 and declined thereafter in both groups (Figure 2). Joinpoint regression identified 1 joinpoint in each group. Among females, the APC was −0.15% from 2018 to 2021 (95% CI: −14.37% to 16.42%) and −23.59% from 2021 to 2024 (95% CI: −39.29% to −3.82%; *P* = 0.037). Among males, the APC was 3.73% from 2018 to 2021 (95% CI: −16.75% to 29.25%) and −17.21% from 2021 to 2024 (95% CI: −35.57% to 6.37%) (Supplemental Figure 1, Supplemental Table 7).

Over the study period, males had higher all-IE mortality than females (age-adjusted RR: 1.24; 95% CI: 1.20-1.29), whereas the sex difference for SU-IE mortality was more modest (age-adjusted RR: 1.11; 95% CI: 1.06-1.16) (Supplemental Table 8).

### Age-stratified trends

Adults aged 35 to 44 years had higher IE-associated mortality throughout the study period. In 2024, this group had 727/1,051 (69.2%) IE-associated deaths (crude mortality rate, 1.60 per 100,000) compared with 324/1,051 (30.8%) deaths (crude mortality rate, 0.70 per 100,000) among adults aged 25 to 34 years.

**Figure 3:**
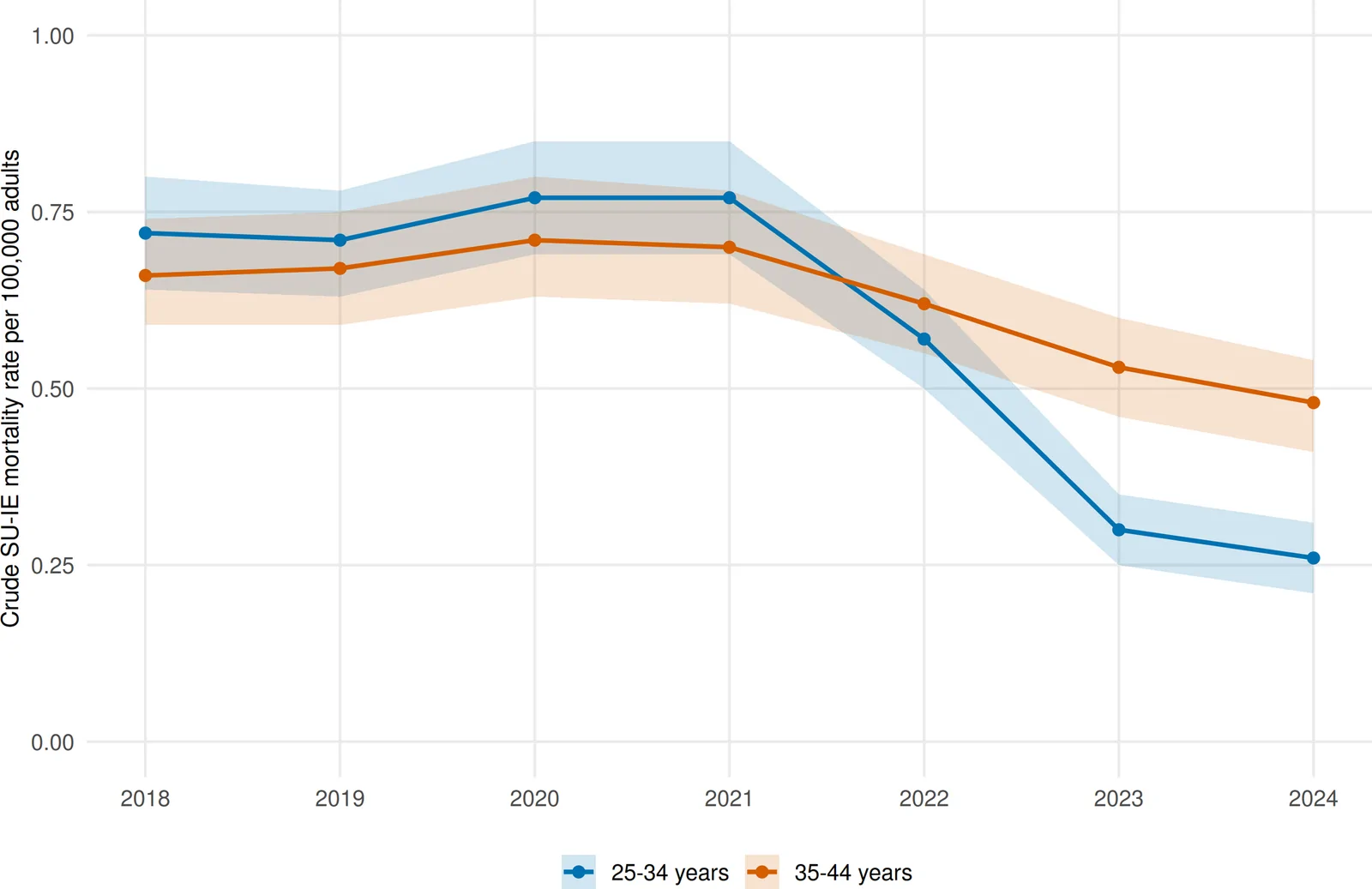
Age-Specific Trends in Substance Use–Associated Infective Endocarditis Mortality Among U.S. Adults, 2018 to 2024 Annual crude mortality rates per 100,000 population are shown for adults aged 25 to 34 years and 35 to 44 years. Points represent annual mortality rates, lines represent temporal trends, and shaded bands indicate 95% CIs. Abbreviation as in Figure 2.

Substance use overlap was consistently higher among adults aged 25 to 34 years, ranging from 330/686 (48.1%) in 2018 to 351/716 (49.0%) in 2021, compared with 274/761 (36.0%) to 304/927 (32.8%) among adults aged 35 to 44 years. After 2021, SU-IE deaths fell sharply among adults aged 25 to 34 years, from 351 in 2021 to 121 in 2024 (65.5% decline), compared with a more modest decline among adults aged 35 to 44 years from 304 to 217 deaths (28.6% decline) (Figure 3). Joinpoint regression identified 1 joinpoint in 2021 for each group. Among adults aged 25 to 34 years, the APC was 3.12% from 2018 to 2021 (95% CI: −21.14% to 34.84%) and −31.73% from 2021 to 2024 (95% CI: −52.71% to −1.45%; *P* = 0.047). Among adults aged 35 to 44 years, the APC was 2.20% from 2018 to 2021 (95% CI: −3.99% to 8.80%) and −12.55% from 2021 to 2024 (95% CI: −18.29% to −6.40%; *P* = 0.014) (Supplemental Figure 2, Supplemental Table 7).

In the expanded adult analysis, adults aged 25 to 44 years accounted for 3,756/5,967 (62.9%) SU-IE deaths, whereas adults aged ≥45 years accounted for 2,211/5,967 (37.1%). Among age groups with reportable annual estimates, the post-2021 decline was most pronounced among adults aged 25 to 34 years, for whom the crude SU-IE mortality rate decreased from 0.77 per 100,000 (95% CI: 0.69-0.85) in 2021 to 0.26 per 100,000 (95% CI: 0.21-0.31) in 2024. Rates also declined among adults aged 35 to 44 years, from 0.70 (95% CI: 0.62-0.78) to 0.48 per 100,000 (95% CI: 0.41-0.54), and among adults aged 45 to 54 years, from 0.49 (95% CI: 0.43-0.56) to 0.33 per 100,000 (95% CI: 0.27-0.38). Rates among adults aged 55 to 64 years remained comparatively stable, changing from 0.29 per 100,000 (95% CI: 0.23-0.34) in 2021 to 0.30 per 100,000 (95% CI: 0.25-0.35) in 2024 (Supplemental Table 2). Annual estimates for adults aged 75 years or older were suppressed because of small death counts and therefore were not displayed in the age-stratified table.

Adults aged 35 to 44 years had higher all-IE mortality than those aged 25 to 34 years (crude RR: 1.49; 95% CI: 1.43-1.55), whereas SU-IE mortality differed only slightly between age groups (crude RR: 1.07; 95% CI: 1.00-1.14) (Supplemental Table 8).

### Race-stratified trends

**Figure 4:**
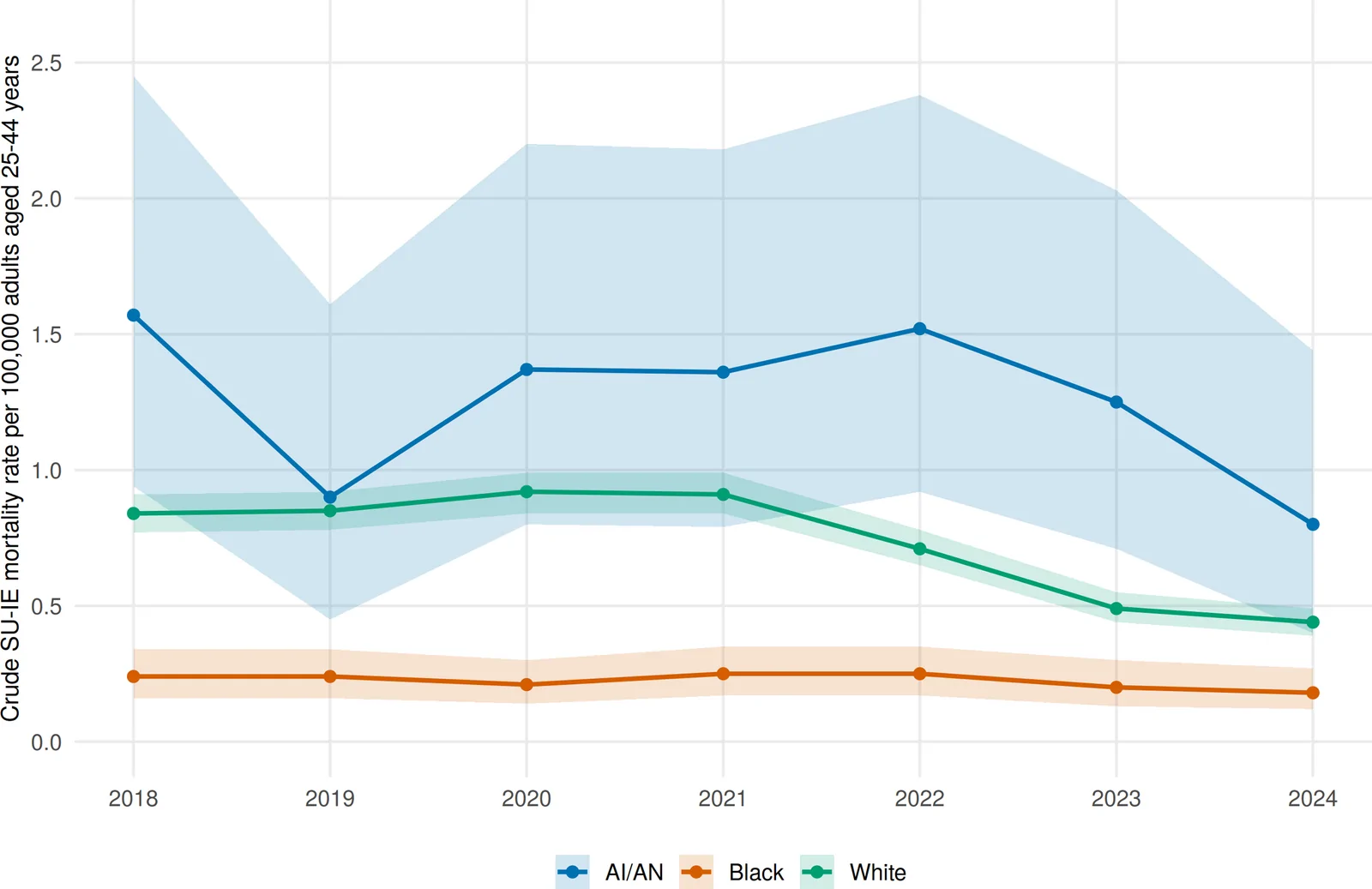
Race-Specific Trends in Substance Use-Associated Infective Endocarditis Mortality Among U.S. Adults Aged 25 to 44 Years, 2018 to 2024 Annual crude mortality rates per 100,000 population are shown for White, Black or African American, and American Indian or Alaska Native adults. Points represent annual mortality rates, lines represent temporal trends, and shaded bands indicate 95% CIs. Other racial groups were not displayed because annual estimates were suppressed or unavailable in multiple years. AI/AN = American Indian or Alaska Native; other abbreviation as in Figure 2.

White adults accounted for the largest number of SU-IE deaths throughout the study period, comprising 540/604 (89.4%) SU-IE deaths in 2018 and 292/338 (86.4%) in 2024. However, American Indian/Alaska Native (AI/AN) adults had the highest crude SU-IE mortality rates across most years, including 1.57 per 100,000 in 2018 and 0.80 per 100,000 in 2024. Black or African-American adults had the lowest rates throughout the study period (Figure 4). Substance use overlap among IE-associated deaths decreased among White adults from 540/1,208 (44.7%) in 2018 to 292/818 (35.7%) in 2024, whereas annual estimates among AI/AN adults varied because of smaller death counts (Supplemental Table 3).

Race-specific Joinpoint analyses were limited to AI/AN, Black or African American, and White adults because other race categories had suppressed or unstable annual counts. Among AI/AN adults, the APC was −4.29% from 2018 to 2024 (95% CI: −15.12% to 7.93%). Among Black or African-American adults, the APC was −2.96% from 2018 to 2024 (95% CI: −8.21% to 2.58%). Among White adults, Joinpoint regression identified 1 joinpoint, with an APC of 2.98% from 2018 to 2021 (95% CI: −14.45% to 23.95%) and −23.46% from 2021 to 2024 (95% CI: −38.82% to −4.25%; *P* = 0.036) (Supplemental Figure 3, Supplemental Table 7).

Compared with White adults, AI/AN adults had the highest all-IE (age-adjusted RR: 1.64; 95% CI: 1.44-1.87) and SU-IE (age-adjusted RR: 1.79; 95% CI: 1.48-2.16) mortality among all racial groups (Supplemental Table 8).

### Urbanization-stratified trends

**Figure 5:**
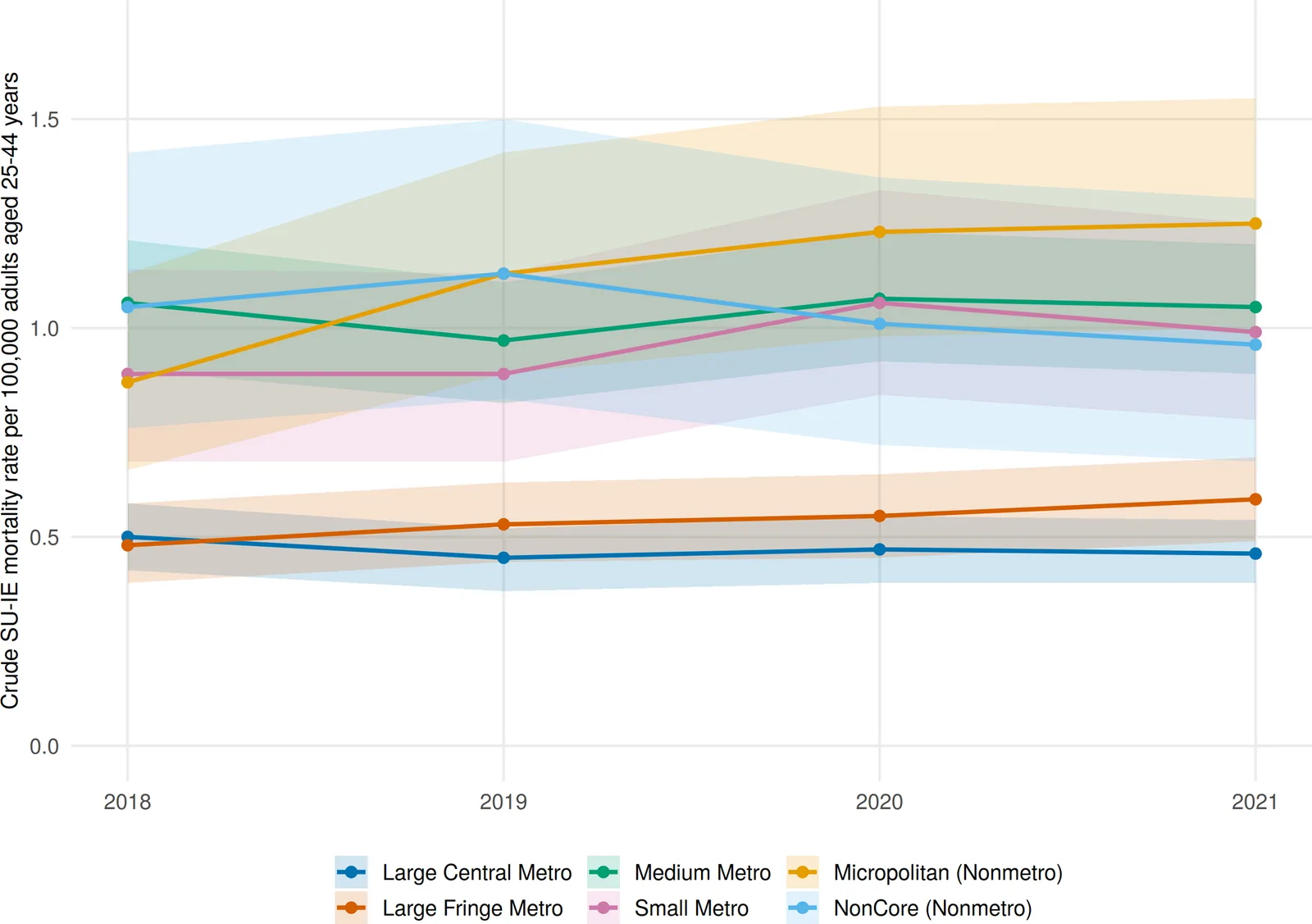
Urbanization-Specific Trends in Substance Use–Associated Infective Endocarditis Mortality Among U.S. Adults Aged 25 to 44 Years, 2018 to 2021 Annual crude mortality rates per 100,000 population are shown across the 2023 National Center for Health Statistics urban-rural classification categories (defined by Office of Management and Budget metropolitan/micropolitan statistical area status and county population size; see Methods): large central metropolitan, large fringe metropolitan, medium metropolitan, small metropolitan, micropolitan nonmetropolitan, and noncore nonmetropolitan counties. Points represent annual mortality rates, lines represent temporal trends, and shaded bands indicate 95% CIs. Rate estimates are limited to 2018 to 2021 because county-level population denominators were unavailable for later years. Abbreviation as in Figure 2.

For years with available population denominators, SU-IE crude mortality rates were higher in medium metropolitan and nonmetropolitan counties than in large central metropolitan counties (Figure 5). In 2021, rates ranged from 0.46 per 100,000 in large central metropolitan counties to 1.25 per 100,000 in micropolitan nonmetropolitan counties (Supplemental Table 4).

During 2018 to 2021, both all-IE and SU-IE mortality rates were substantially higher in nonmetropolitan than in large central metropolitan counties. Compared with large central metropolitan counties, the highest crude SU-IE mortality was observed in micropolitan counties (RR: 2.39; 95% CI: 2.08-2.75), with similarly elevated rates in noncore counties (Supplemental Table 8).

### State-level variation

**Figure 6:**
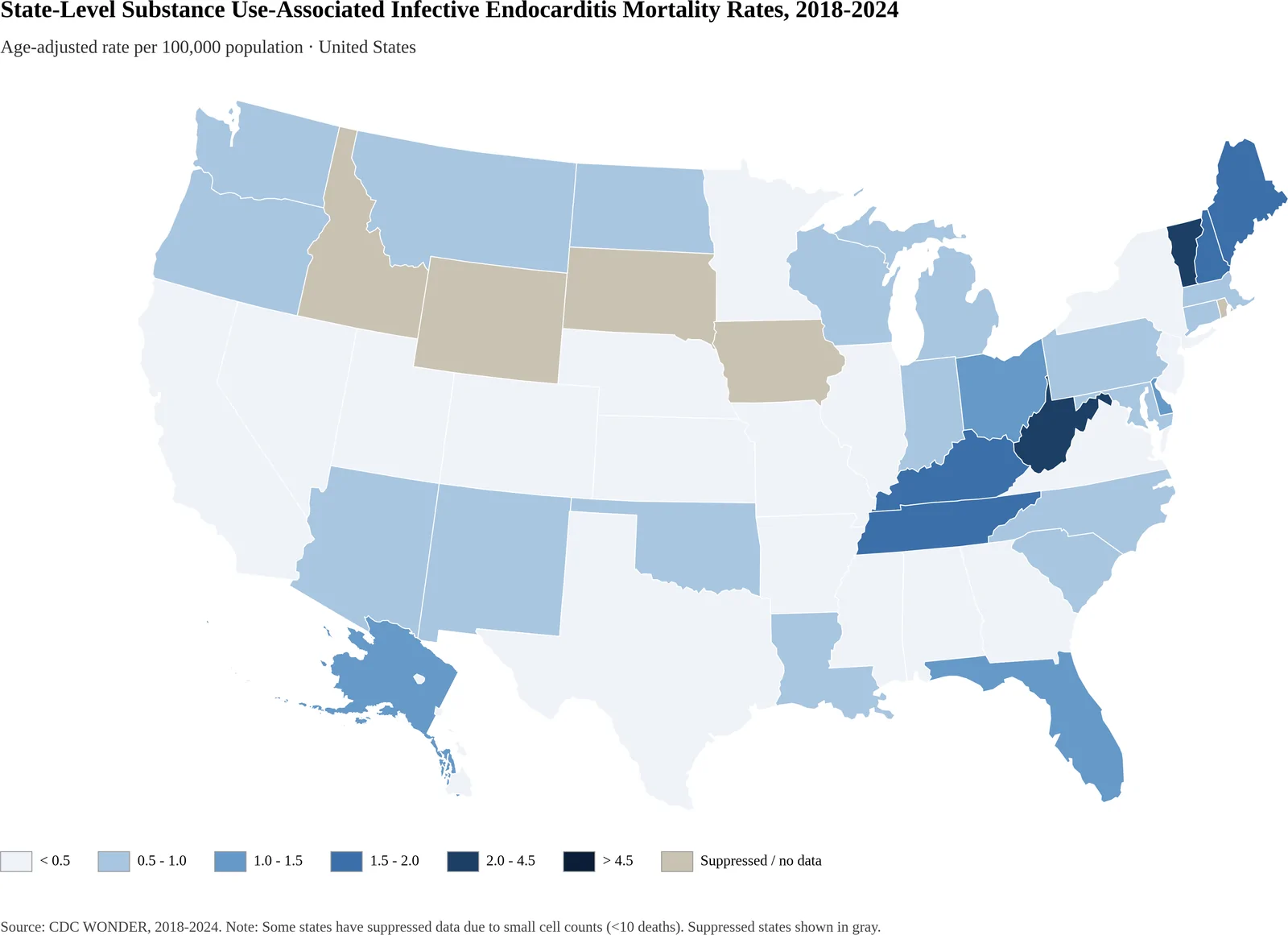
State-Level Substance Use–Associated Infective Endocarditis Mortality Rates Among U.S. Adults Aged 25 to 44 Years, 2018 to 2024 The choropleth map displays pooled age-adjusted mortality rates per 100,000 population for substance use–associated infective endocarditis during 2018 to 2024. Darker blue shading indicates higher mortality rates. States with suppressed or unavailable estimates are shown in gray; estimates were suppressed when death counts did not meet CDC WONDER reliability or confidentiality requirements. Data source: CDC WONDER Multiple Cause of Death Files, 2018 to 2024.

Age-adjusted state-level SU-IE mortality rates varied geographically, with the highest rates concentrated in Appalachia and parts of New England (Figure 6). The highest age-adjusted mortality rates were observed in West Virginia (4.17 per 100,000), Vermont (2.89), Tennessee (1.93), and Kentucky (1.88).

SU-IE death counts decreased across all urban-rural categories from 2021 to 2024. The greatest relative declines occurred in small metropolitan counties, where deaths decreased by 63.9%, and micropolitan counties, where deaths decreased by 60.2%. Medium metropolitan counties experienced the greatest absolute decline, with 103 fewer deaths in 2024 than in 2021 (Supplemental Table 5).

### Sensitivity analyses

Sensitivity analyses yielded findings consistent with the primary analysis. After excluding cannabinoid-related codes, total adult SU-IE deaths changed minimally, from 5,967 to 5,957. The temporal pattern was unchanged, with deaths increasing to 1,023 in 2021 and declining to 641 in 2024. The age-adjusted mortality rate declined from 0.50 per 100,000 in 2021 to 0.30 per 100,000 in 2024 (Supplemental Table 6).

In the opioid-specific analysis, 617 opioid-associated IE deaths were identified among adults aged ≥25 years. Deaths increased from 85 in 2018 to 108 in 2021 and then declined to 65 in 2024. The age-adjusted mortality rate declined from 0.07 per 100,000 in 2021 to 0.03 per 100,000 in 2024. The peak-and-decline pattern therefore persisted after exclusion of cannabinoid-related codes and was also present in the opioid-specific analysis (Supplemental Table 6).

## Discussion

In this national analysis of U.S. adults aged 25 to 44 years, substance use was documented in more than one-third of IE-associated deaths from 2018 through 2024. IE-associated and SU-IE mortality were stable to increase through 2020 to 2021 and declined thereafter, with the proportion of IE deaths overlapping with substance use decreasing from >40% in 2018 to approximately 32% in 2023 to 2024. In an expanded analysis of adults aged ≥25 years, SU-IE mortality remained concentrated among younger adults, although adults aged ≥45 years accounted for more than one-third of adult SU-IE deaths. Sensitivity analyses excluding cannabinoid-related codes and restricting the definition to opioid-related codes yielded similar peak-and-decline patterns (Central Illustration).

**Central Illustration:**
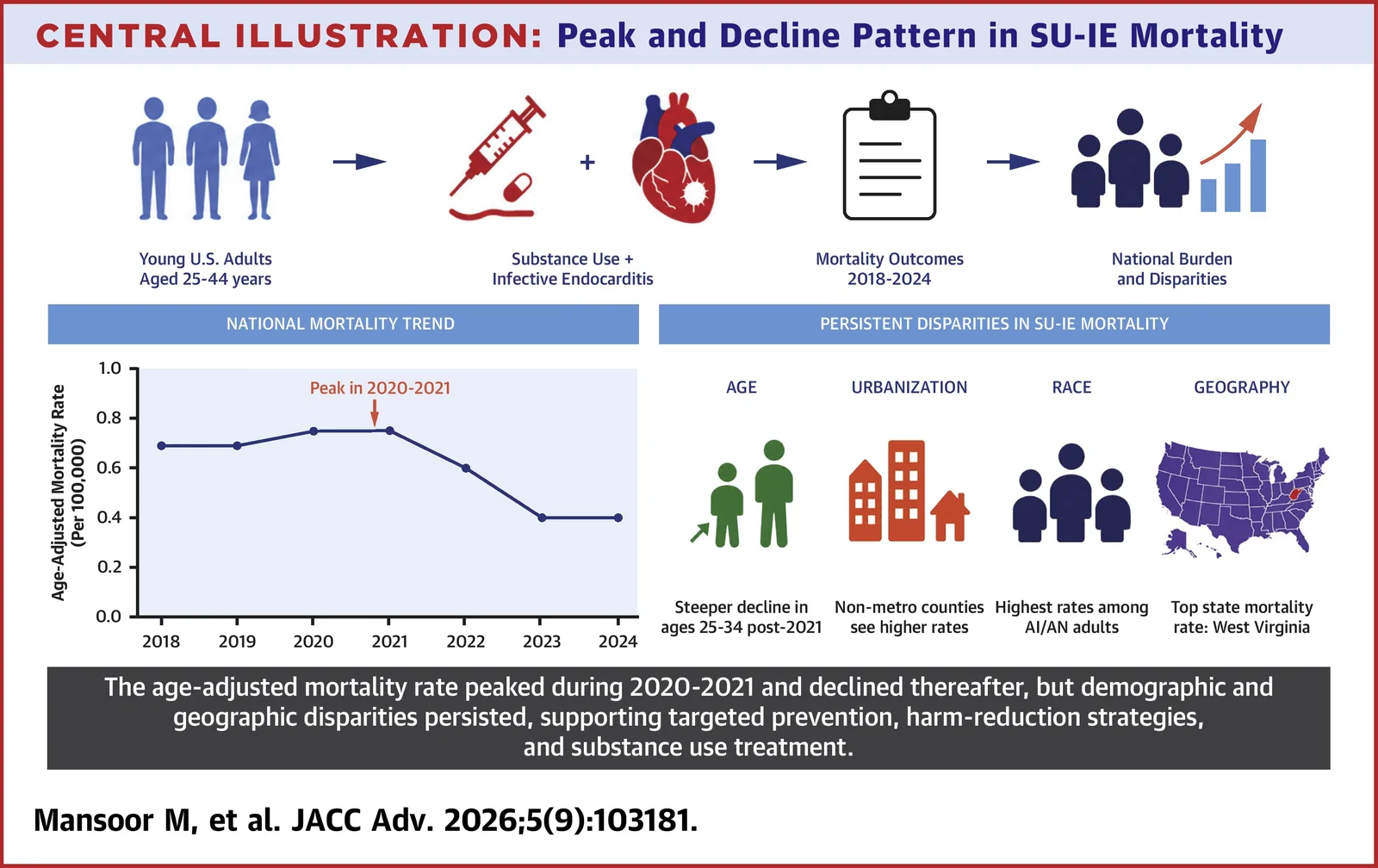
Peak and Decline Pattern in SU-IE Mortality This graphical summary presents national substance use–associated infective endocarditis (SU-IE) mortality among U.S. adults aged 25 to 44 years using CDC WONDER Multiple Cause of Death data from 2018 to 2024. SU-IE accounted for 3,756 deaths, representing 38.5% of all IE-associated deaths. Mortality peaked in 2020 to 2021 and declined thereafter, although substantial demographic and geographic disparities persisted. AI/AN = American Indian or Alaska Native.

### Postpandemic mortality pattern

Our findings extend prior national reports by demonstrating that the previously rising trajectory of SU-IE mortality among young adults did not persist through the most contemporary period. Instead, mortality peaked around 2020 to 2021 and declined thereafter, with both death counts and mortality rates decreasing through 2024. Earlier CDC Multiple Cause-of-Death studies generally documented increasing IE-associated or SU-IE mortality among younger adults and persons with injection drug use–related indicators. In contrast, the present analysis extends national surveillance through 2024 and identifies a peak during 2020 to 2021 followed by declining death counts and mortality rates through 2024 (Table 2). Joinpoint regression supported a post-2021 inflection, showing a transition from relative stability or modest increases before 2021 to declining mortality rates after 2021. These findings suggest a contemporary shift in recorded SU-IE mortality after the early pandemic period, while recognizing that mortality data cannot determine whether this reflects changes in IE incidence, survival, competing mortality, or coding practices.

**Table 2:** Comparison of the Current Study With Previous CDC Multiple Cause-of-Death Studies of Infective Endocarditis Mortality

First Author	Database	Years	Population	Substance-Use/IE Definition	Key Findings	Added Value of Current Study
Abdul Jabbar et al^4^	CDC WONDER Multiple Cause-of-Death	1999-2022	Young U.S. adults	IE-associated deaths with substance-abuse overlap	Found that substance abuse accounted for approximately one-third of IE-related deaths in young adults, with increasing rates over time	Current study adds 2023-2024 data, identifies a post-2021 decline, and evaluates demographic and geographic disparities
Chobufo et al^6^	CDC WONDER Multiple Cause-of-Death	1999-2020	U.S. decedents; age-stratified analyses including adults aged 25-44 years	IE-associated mortality using multiple-cause-of-death ICD-10 codes	Reported increasing IE mortality among younger adults despite declining IE mortality in the overall population	Current study focuses specifically on SU-IE mortality from 2018-2024 and includes sensitivity analyses
Njoroge et al^9^	CDC Multiple Cause-of-Death	1999-2016	Persons who inject drugs	IE mortality among persons with injection drug use-related indicators	Demonstrated rising IE mortality among persons who inject drugs, particularly among younger adults	Current study extends analysis through 2024 and evaluates the contemporary post-2021 decline
Current study	CDC WONDER Multiple Cause-of-Death, Single Race Results	2018-2024	U.S. adults aged 25-44 years; expanded analysis among adults aged ≥25 years	IE codes I33.0, I33.9, I38 with co-listed F11, F12, F13, F14, F15, or F19 codes	SU-IE mortality peaked in 2020-2021 and declined through 2024, with persistent demographic and geographic disparities	Provides the most contemporary national mortality analysis and includes ≥45-year, cannabinoid-exclusion, and opioid-specific sensitivity analyses

The studies differed in their observation periods, age groups, and definitions of substance use-associated or injection drug use-associated IE; therefore, their estimates should not be interpreted as directly comparable.

CDC = Centers for Disease Control and Prevention; WONDER = Wide-ranging Online Data for Epidemiologic Research; other abbreviations as in Table 1.

The post-2021 decline should therefore be interpreted cautiously. National overdose mortality also declined during the later study period. From 2023 to 2024, the age-adjusted drug overdose death rate decreased by 26.2%, from 31.3 to 23.1 deaths per 100,000 standard population.^10^ Although national drug overdose mortality also declined during the same period, these parallel trends should not be interpreted as evidence that decreases in recorded SU-IE mortality necessarily reflect reductions in substance-related morbidity, because mortality data cannot distinguish changes in disease incidence, survival, competing mortality, or death certificate coding.

Surveillance and coding factors may also contribute to the observed pattern. CDC WONDER mortality data are based on death certificates, and substance use codes depend on whether substance use was documented by the certifier as a contributing condition.^5^ Changes in death certificate coding practices, toxicology testing, medical examiner involvement, and documentation of substance use may influence apparent trends. Therefore, the observed decline should be interpreted as a decline in recorded SU-IE mortality, not definitive evidence of a decline in injection-associated IE incidence.

### Sex differences

Males had higher absolute IE mortality, yet females had higher proportional substance use overlap among IE deaths. This distinction has important implications: although young men carry a larger aggregate IE burden, the relative contribution of substance use to IE mortality was more pronounced among young women throughout the study period. Sex-specific differences in injection practices, stigma, intimate partner dynamics, social vulnerability, and access to addiction treatment may partly explain this pattern. Prior studies have shown that women who inject drugs experience distinct social and treatment-related barriers, and recent work has highlighted sex differences in drug use–associated IE.^11^^,^^12^ In our analysis, the post-2021 decline in SU-IE mortality was statistically significant among females but not males, warranting further mechanistic investigation.

### Age differences

Adults aged 25 to 34 years had higher substance use overlap among IE deaths, whereas adults aged 35 to 44 years had higher absolute IE mortality rates, suggesting different risk patterns within the young-adult age range. The sharp post-2021 decline among adults aged 25 to 34 years (APC: −31.73%) was among the most striking findings. This group may be more sensitive to changes in substance use patterns, harm-reduction availability, and competing overdose mortality.

The more modest decline among adults aged 35 to 44 years may reflect a more heterogeneous mix of IE risk factors beyond substance use, including structural valve disease, comorbidities, and health care–associated IE. In the expanded adult analysis, adults aged ≥45 years accounted for more than one-third of adult SU-IE deaths, supporting consideration of SU-IE beyond young adults alone.

### Racial disparities

White adults accounted for the largest absolute SU-IE burden, whereas AI/AN adults had the highest population-adjusted rates throughout the study period. AI/AN adults had crude SU-IE rates more than twice those of White adults in most years, despite smaller absolute counts. This rate-count dichotomy is essential for public health resource allocation: high absolute counts may guide allocation across populous communities, whereas high rates identify populations experiencing disproportionate mortality risk.

Contributors to elevated AI/AN rates likely include rurality, limited access to addiction treatment and specialty cardiovascular care, structural social determinants of health, and differential burden of polysubstance use.^13^ Black adults had lower documented SU-IE rates and lower overlap proportions than White and AI/AN adults throughout the study, which may reflect true differences in IE risk pathways, differential documentation of substance use on death certificates, or unmeasured confounders.

### Urbanization and geographic disparities

SU-IE mortality rates were higher in medium metropolitan and nonmetropolitan counties than in large central metropolitan counties, suggesting that SU-IE mortality is not confined to large urban centers. Geographic patterns were presented descriptively, with the highest state-level rates concentrated in Appalachia and parts of New England.

### Clinical and public health implications

These findings support closer integration of IE prevention and management with substance-use treatment, particularly for populations with persistent disparities.^14^^,^^15^ The observed differences by sex, age, race, and urbanization may help identify groups that could benefit from earlier recognition, multidisciplinary care, and improved linkage to addiction treatment and follow-up care.

### Study Limitations

This study has several important limitations. First, CDC WONDER data are derived from death certificates and subject to misclassification and coding variability across states, counties, and time. Second, our SU-IE definition uses F-code ICD-10 substance use diagnoses, which do not specifically identify injection drug use; we therefore use the term “substance use–associated” rather than “injection drug use–associated”. Third, clinical details, including microbiology, valve type, surgery, antibiotic therapy, recurrent IE, housing status, and access to addiction treatment are unavailable. Fourth, multiple substances may be listed on a death certificate, and substance classes were not mutually exclusive. Fifth, urbanization-specific population denominators were unavailable in the extracted output for 2022 to 2024, limiting rate-based comparisons for those years. Sixth, race misclassification on death certificates may affect race-stratified estimates, and Hispanic origin was not incorporated in the primary race analysis. Seventh, several race and state subgroup estimates were suppressed due to small counts. Finally, this is a descriptive ecological analysis and cannot establish causality.

## Conclusions

Among U.S. adults aged 25 to 44 years, substance use was documented in more than one-third of IE-associated deaths from 2018 through 2024, accounting for 3,756 deaths. Although IE-associated and SU-IE mortality peaked during 2020 to 2021 and declined thereafter, persistent disparities by sex, age, race, urbanization, and geography remained evident. Expanded adult analyses showed that SU-IE mortality was not limited to young adults, although sensitivity analyses excluding cannabinoid-related codes and restricting to opioid-related codes supported the robustness of the observed peak-and-decline pattern. These findings underscore SU-IE as a continuing and potentially preventable contributor to premature cardiovascular and infectious mortality. Improved surveillance and integrated cardiovascular, infectious disease, and substance-use care are needed to better identify high-risk populations and reduce preventable SU-IE mortality.

Perspectives **COMPETENCY IN SYSTEMS-BASED PRACTICE:** SU-IE remains an important contributor to premature mortality among young U.S. adults, with persistent disparities by sex, age, race, urbanization, and geography despite declining recorded mortality after 2021.

**TRANSLATIONAL OUTLOOK:** Future studies using linked clinical, hospitalization, microbiology, surgical, and addiction-treatment data are needed to determine whether declining recorded SU-IE mortality reflects changes in incidence, survival, competing overdose mortality, or death certificate coding.

## Funding support and author disclosures

The authors have reported that they have no relationships relevant to the contents of this paper to disclose.
