# Supplementary material for: Substance Use–Associated Infective Endocarditis Mortality in Young U.S. Adults, 2018 to 2024
Source: JACC Adv. 2026 Sep 23;5(9):103181. doi: 10.1016/j.jacadv.2026.103181 (PMC13616512; doi:10.1016/j.jacadv.2026.103181)
Supplement: Supplementary file 1 [file mmc1.pdf]

SUPPLEMENTAL FIGURES

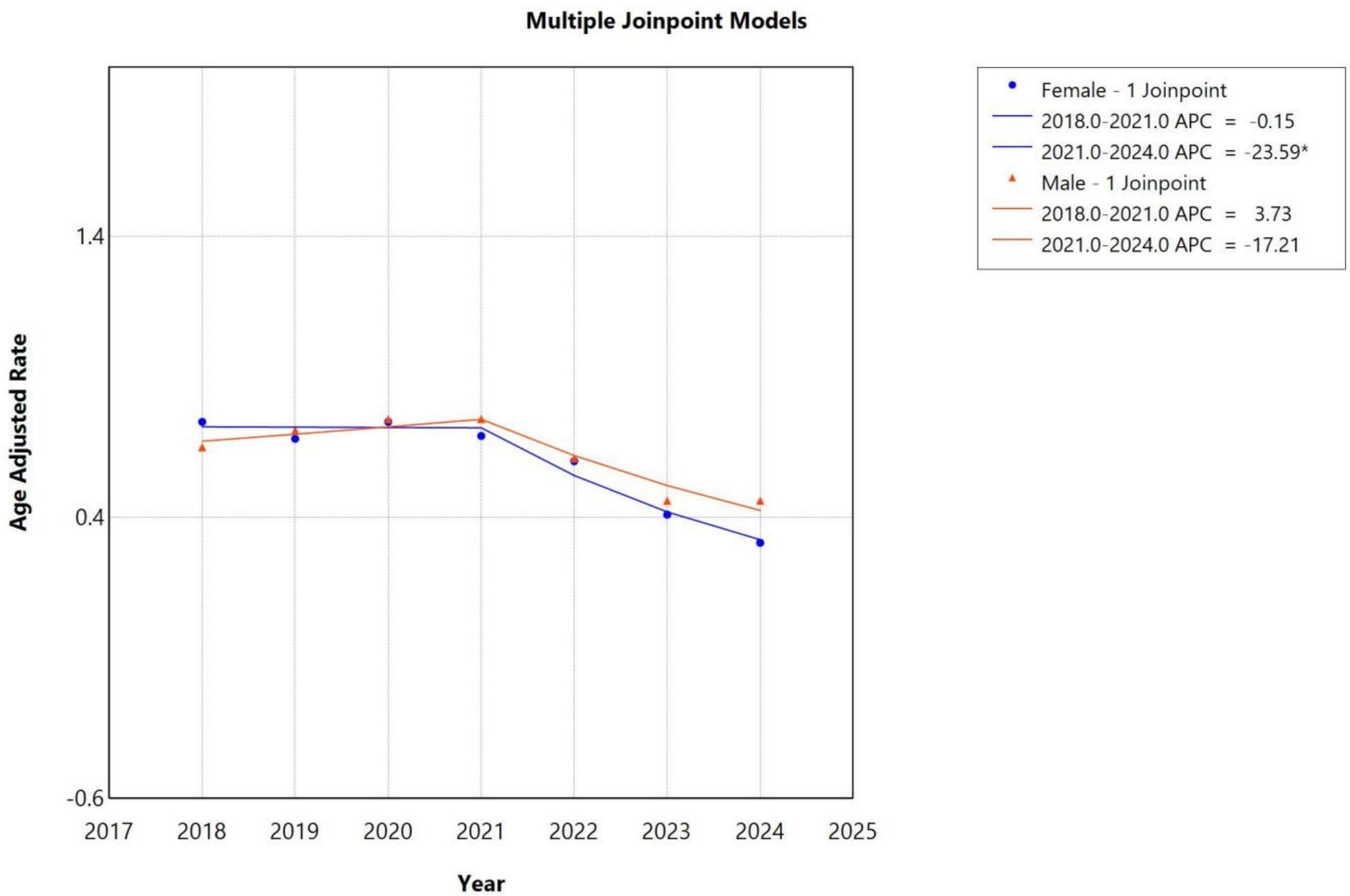

Supplemental Figure 1. Joinpoint Regression of Sex-Specific Substance Use-Associated Infective Endocarditis Mortality Rates Among U.S. Adults Aged 25-44 Years, 2018-2024. Joinpoint regression of annual age-adjusted substance use–associated infective endocarditis (SU-IE) mortality rates per 100,000 population among males and females. Solid lines represent fitted Joinpoint regression models, and the dashed vertical line indicates the identified joinpoint in 2021. Among females, the annual percent change (APC) was −0.15% from 2018 to 2021 and −23.59% from 2021 to 2024 ($P<0.05$). Among males, the APC was 3.73% from 2018 to 2021 and −17.21% from 2021 to 2024. Asterisks denote statistically significant APCs.

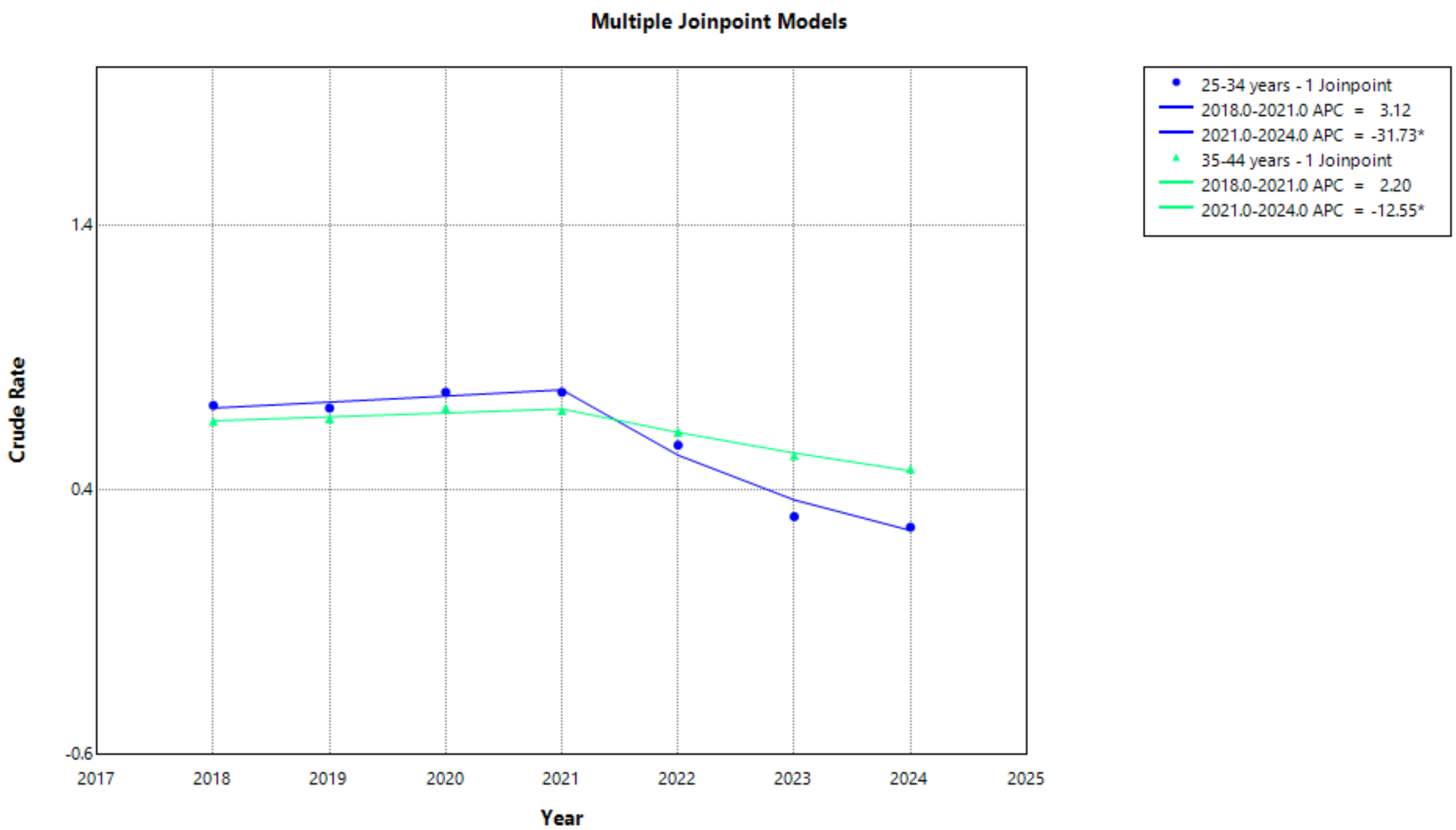

Supplemental Figure 2. Joinpoint Regression of Age-Specific Substance Use-Associated Infective Endocarditis Mortality Rates Among U.S. Adults Aged 25–44 Years, 2018–2024. Joinpoint regression of annual crude substance use–associated infective endocarditis (SU-IE) mortality rates per 100,000 population among adults aged 25–34 years and 35–44 years. Solid lines represent fitted Joinpoint regression models, and the dashed vertical line indicates the identified joinpoint in 2021. Among adults aged 25–34 years, the annual percent change (APC) was 3.12% from 2018 to 2021 and −31.73% from 2021 to 2024 (*P*=.047). Among adults aged 35–44 years, the APC was 2.20% from 2018 to 2021 and −12.55% from 2021 to 2024 (*P*=.014). Asterisks denote statistically significant APCs.

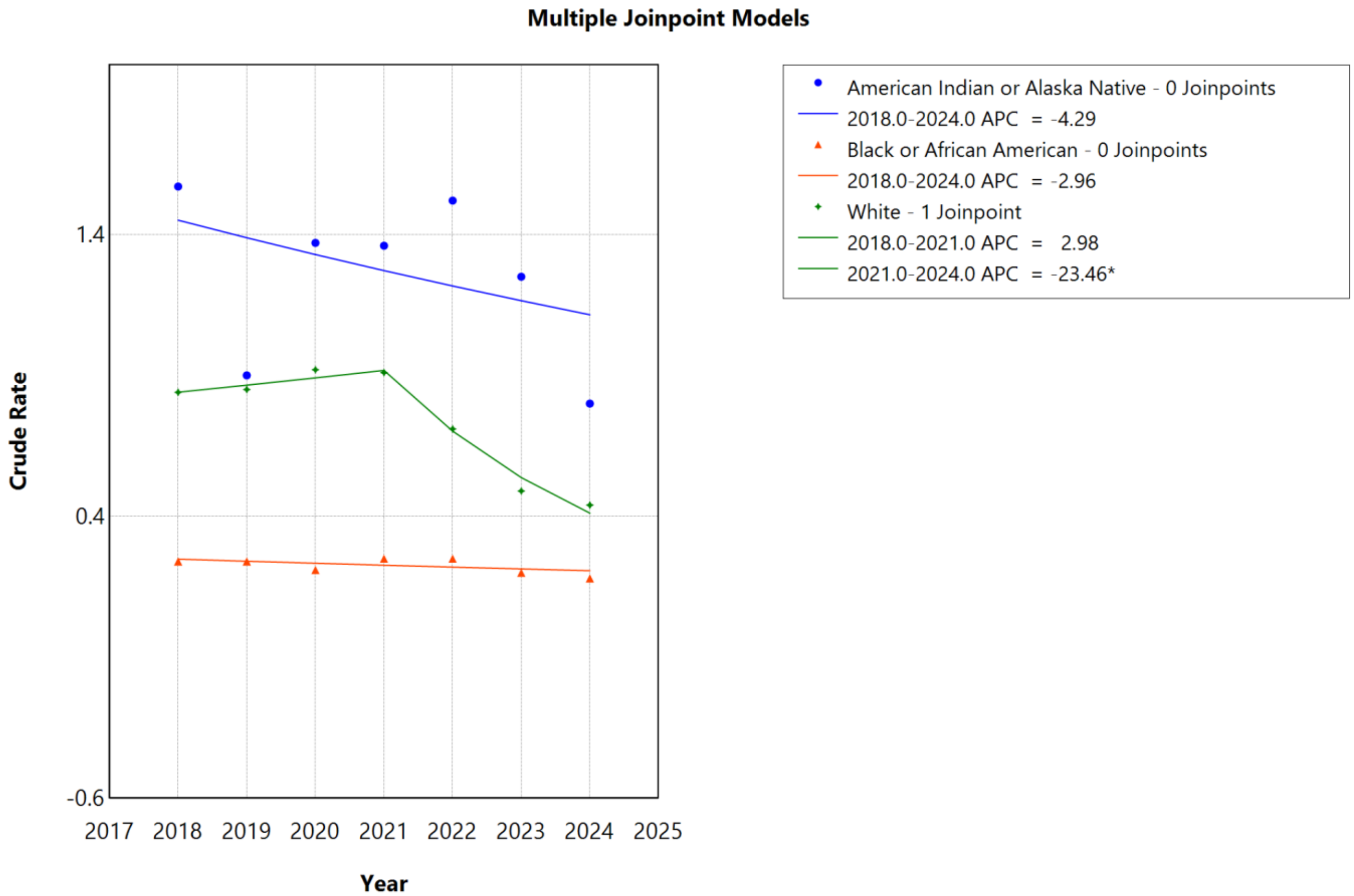

Supplemental Figure 3. Joinpoint Regression of Race-Specific Substance Use-Associated Infective Endocarditis Mortality Rates Among U.S. Adults Aged 25–44 Years, 2018–2024. Joinpoint regression of annual crude substance use–associated infective endocarditis (SU-IE) mortality rates per 100,000 population among White, Black or African American, and American Indian or Alaska Native (AI/AN) adults. Solid lines represent fitted Joinpoint regression models, and the dashed vertical line indicates the identified joinpoint in 2021 for White adults. Among White adults, the annual percent change (APC) was 2.98% from 2018 to 2021 and −23.46% from 2021 to 2024 (P=.036). No joinpoint was identified for Black or African American adults (APC, −2.96% for 2018–2024) or American Indian or Alaska Native adults (APC, −4.29% for 2018–2024). Asterisks denote statistically significant APCs.

SUPPLEMENTAL TABLES

Supplemental Table 1. Substance-Use Overlap Among Infective Endocarditis Deaths by Sex, United States, 2018-2024

Year	Female Total IE Deaths	Female Substance-Use IE Deaths	Female Overlap, %	Male Total IE Deaths	Male Substance-Use IE Deaths	Male Overlap, %
2018	701	328	46.8	746	276	37.0
2019	637	302	47.4	745	302	40.5
2020	737	326	44.2	867	329	37.9
2021	743	311	41.9	900	344	38.2
2022	653	261	40.0	796	268	33.7
2023	505	171	33.9	664	200	30.1
2024	414	139	33.6	637	199	31.2

Substance-use overlap was calculated as the number of SU-IE deaths divided by the total number of IE-associated deaths within each sex and calendar year. **Abbreviations:** IE, infective endocarditis; SU-IE, substance use–associated infective endocarditis.

Supplemental Table 2. Age-Stratified Substance Use–Associated Infective Endocarditis Mortality Among U.S. Adults Aged ≥25 years

Year	25-34 Years, Deaths	Rate (95% CI)	35-44 Years, Deaths	Rate (95% CI)	45-54 Years, Deaths	Rate (95% CI)	55-64 Years, Deaths	Rate (95% CI)	65-74 Years, Deaths	Rate (95% CI)
2018	330	0.72 (0.64-0.80)	274	0.66 (0.59-0.74)	167	0.40 (0.34-0.46)	90	0.21 (0.17-0.26)	25	0.08 (0.05-0.12)
2019	325	0.71 (0.63-0.78)	279	0.67 (0.59-0.75)	184	0.45 (0.39-0.52)	103	0.24 (0.20-0.29)	19	0.06 (0.04-0.09)
2020	354	0.77 (0.69-0.85)	301	0.71 (0.63-0.80)	175	0.43 (0.37-0.50)	105	0.25 (0.20-0.29)	21	0.06 (0.04-0.10)
2021	351	0.77 (0.69-0.85)	304	0.70 (0.62-0.78)	201	0.49 (0.43-0.56)	122	0.29 (0.23-0.34)	39	0.12 (0.08-0.16)
2022	258	0.57 (0.50-0.64)	271	0.62 (0.55-0.69)	163	0.40 (0.34-0.47)	105	0.25 (0.20-0.30)	48	0.14 (0.10-0.19)
2023	135	0.30 (0.25-0.35)	236	0.53 (0.46-0.60)	124	0.31 (0.25-0.36)	138	0.33 (0.27-0.38)	37	0.11 (0.08-0.15)
2024	121	0.26 (0.21-0.31)	217	0.48 (0.41-0.54)	134	0.33 (0.27-0.38)	125	0.30 (0.25-0.35)	39	0.11 (0.08-0.15)
**Total**	**1,874**	—	**1,882**	—	**1,148**	—	**788**	—	**228**	—

Death counts and crude mortality rates are shown for reportable 10-year age groups. Rates are expressed per 100,000 population within each age group. Results for adults aged 75 to 84 years and 85 years or older were suppressed by CDC WONDER because of small annual death counts and are therefore not displayed. Consequently, the age-group totals in this table do not equal the overall number of SU-IE deaths among adults aged ≥25 years. An em dash indicates that a pooled period rate was not calculated. **Abbreviations:** CDC, Centers for Disease Control and Prevention; IE, infective endocarditis; SU-IE, substance use–associated infective endocarditis; WONDER, Wide-ranging Online Data for Epidemiologic Research.

Supplemental Table 3. Substance-Use Overlap Among Infective Endocarditis Deaths by Race, United States, 2018-2024

Year	AI/AN Total IE Deaths	AI/AN Substance-Use IE Deaths	AI/AN Overlap, %	Black Total IE Deaths	Black Substance-Use IE Deaths	Black Overlap, %	White Total IE Deaths	White Substance-Use IE Deaths	White Overlap, %
2018	32	19	59.4	160	30	18.8	1208	540	44.7
2019	25	11	44.0	150	30	20.0	1163	549	47.2
2020	47	17	36.2	179	27	15.1	1331	597	44.9
2021	42	17	40.5	186	32	17.2	1381	598	43.3
2022	36	19	52.8	200	33	16.5	1182	466	39.4
2023	32	16	50.0	171	27	15.8	925	323	34.9
2024	32	11	34.4	170	25	14.7	818	292	35.7

Substance-use overlap was calculated as the number of SU-IE deaths divided by the total number of IE-associated deaths within each racial group and calendar year. Categories reflect the CDC WONDER Single Race classification. Data for racial groups with sparse or suppressed annual counts are not displayed in this table. **Abbreviations:** AI/AN, American Indian or Alaska Native; IE, infective endocarditis; SU-IE, substance use–associated infective endocarditis.

Supplemental Table 4. Crude Substance Use-Associated Infective Endocarditis Mortality Rates by Urban-Rural Classification, United States, 2018-2021

Year	Large Central Metro	Large Fringe Metro	Medium Metro	Small Metro	Micropolitan	Noncore
2018	0.50	0.48	1.06	0.89	0.87	1.05
2019	0.45	0.53	0.97	0.89	1.13	1.13
2020	0.47	0.55	1.07	1.06	1.23	1.01
2021	0.46	0.59	1.05	0.99	1.25	0.96

Mortality rates are expressed per 100,000 population and were calculated using annual population estimates for each county urban-rural category. Counties were classified according to the 2023 National Center for Health Statistics Urban-Rural Classification Scheme for Counties as large central metropolitan, large fringe metropolitan, medium metropolitan, small metropolitan, micropolitan nonmetropolitan, or noncore nonmetropolitan. Rate estimates were limited to 2018–2021 because corresponding population denominators by urbanization category were not available in the selected CDC WONDER dataset after 2021. **Abbreviations:** CDC, Centers for Disease Control and Prevention; NCHS, National Center for Health Statistics; SU-IE, substance use–associated infective endocarditis.

Supplemental Table 5. Absolute and Percent Change in Substance Use-Associated Infective Endocarditis Deaths by Urbanization Level, United States, 2021-2024

Urbanization	2021 Deaths, No.	2024 Deaths, No.	Absolute Change, No.	Percent Change, %
Large Central Metro	141	94	−47	−33.3
Large Fringe Metro	130	71	−59	−45.4
Medium Metro	185	82	−103	−55.7
Small Metro	72	26	−46	−63.9
Micropolitan	83	33	−50	−60.2
Noncore	39	26	−13	−33.3

Absolute change was calculated as the number of deaths in 2024 minus the number in 2021. Relative change was calculated as the absolute change divided by the 2021 number of deaths and multiplied by 100. Counts are presented for 2021 and 2024; mortality rates were not calculated after 2021 because population denominators by urban-rural category were unavailable in the selected dataset. County categories were defined using the 2023 NCHS Urban-Rural Classification Scheme for Counties. **Abbreviations:** NCHS, National Center for Health Statistics; SU-IE, substance use–associated infective endocarditis.

Supplemental Table 6. Sensitivity Analyses of Substance Use-Associated Infective Endocarditis Mortality Among U.S. Adults Aged 25 Years or Older, 2018-2024

Analysis	Total deaths	2021 deaths	2024 deaths	2021 age-adjusted rate	2024 age-adjusted rate
Primary SU-IE, adults aged >=25 years	5,967	1,024	642	0.50	0.30
Excluding cannabinoid-related codes	5,957	1,023	641	0.50	0.30
Opioid-specific analysis	617	108	65	0.07	0.03

Mortality rates are expressed per 100,000 population and were age-standardized to the 2000 U.S. standard population. The cannabinoid-exclusion analysis omitted deaths identified solely through cannabinoid-related diagnosis codes. The opioid-specific analysis was restricted to deaths with an opioid-related diagnosis code. **Abbreviations:** IE, infective endocarditis; SU-IE, substance use–associated infective endocarditis.

Supplemental Table 7. Joinpoint Regression of Substance Use-Associated Infective Endocarditis Mortality Rates Among U.S. Adults Aged 25 to 44 Years, 2018-2024

Population	Joinpoint	APC, 2018-2021, % (95% CI)	APC, 2021-2024, % (95% CI)	AAPC, 2018-2024, % (95% CI)	AAPC *P* Value
Overall	2021	2.01 (−23.59 to 36.18)	−20.33 (−43.55 to 12.44)	−9.85 (−18.62 to −0.13)	.047
Male	2021	3.73 (−16.75 to 29.25)	−17.21 (−35.57 to 6.37)	−7.33 (−14.11 to −0.02)	.049
Female	2021	−0.15 (−14.37 to 16.42)	−23.59 (−39.29 to −3.82)	−12.65 (−17.99 to −6.97)	<.001
Age 25-34 years	2021	3.12 (−21.14 to 34.84)	−31.73 (−52.71 to −1.45)	−16.10 (−24.35 to −6.94)	<.001
Age 35-44 years	2021	2.20 (−3.99 to 8.80)	−12.55 (−18.29 to −6.40)	−5.46 (−7.43 to −3.45)	<.001
White	2021	2.98 (−14.45 to 23.95)	−23.46 (−38.82 to −4.25)	−11.22 (−16.91 to −5.15)	<.001
Black	None	—	—	−2.96 (−8.21 to 2.58)†	.222
American Indian or Alaska Native	None	—	—	−4.29 (−15.12 to 7.93)†	.391

Joinpoint regression was performed using a grid-search method permitting 0 or 1 joinpoint, with at least 2 observations between joinpoints. Statistical significance was assessed using Monte Carlo permutation testing with 4,499 permutations and a 2-sided α of 0.05. Known standard errors obtained from the annual mortality rates were used, and errors were assumed to be uncorrelated. Bold values indicate statistical significance at P<0.05. When no joinpoint was identified, a single APC was estimated for the entire 2018–2024 period and is presented in the AAPC column. **Abbreviations:** AAPC, average annual percent change; AI/AN, American Indian or Alaska Native; APC, annual percent change; CI, confidence interval; SU-IE, substance use–associated infective endocarditis.

Supplemental Table 8. Crude and Age-Adjusted Mortality Rate Ratios for Infective Endocarditis and Substance Use-Associated Infective Endocarditis, United States, 2018-2024

Outcome	Comparison	Reference Group	Crude RR (95% CI)	Age-Adjusted RR (95% CI)
**Sex**				
All IE	Male	Female	1.20 (1.15-1.24)	1.24 (1.20-1.29)
SU-IE	Male	Female	1.02 (0.96-1.09)	1.11 (1.06-1.16)
**Age Group***				
All IE	35-44 years	25-34 years	1.49 (1.43-1.55)	NA
SU-IE	35-44 years	25-34 years	1.07 (1.00-1.14)	NA
**Race**				
All IE	American Indian or Alaska Native	White	1.59 (1.40-1.81)	1.64 (1.44-1.87)
All IE	Black or African American	White	0.76 (0.72-0.81)	0.80 (0.75-0.85)
All IE	Asian	White	0.12 (0.10-0.15)	0.12 (0.10-0.14)
All IE	Native Hawaiian or Other Pacific Islander	White	0.90 (0.63-1.29)	0.89 (0.61-1.29)
All IE	More than one race	White	0.50 (0.42-0.58)	0.51 (0.43-0.61)
SU-IE	American Indian or Alaska Native	White	1.69 (1.40-2.05)	1.79 (1.48-2.16)
SU-IE	Black or African American	White	0.30 (0.26-0.35)	0.36 (0.30-0.42)
SU-IE	Asian	White	0.05 (0.03-0.08)	Not estimable†
SU-IE	More than one race	White	0.45 (0.35-0.59)	0.43 (0.31-0.59)
SU-IE	Native Hawaiian or Other Pacific Islander	White	Not reportable‡	Not reportable‡
**Urbanization (2018-2021 only)**				
All IE	Large Fringe Metro	Large Central Metro	1.11 (1.03-1.20)	Not applicable
All IE	Medium Metro	Large Central Metro	1.84 (1.72-1.98)	Not applicable
All IE	Small Metro	Large Central Metro	1.87 (1.71-2.06)	Not applicable
All IE	Micropolitan (Nonmetro)	Large Central Metro	2.18 (1.99-2.38)	Not applicable

All IE	NonCore (Nonmetro)	Large Central Metro	2.40 (2.16-2.66)	Not applicable
SU-IE	Large Fringe Metro	Large Central Metro	1.15 (1.02-1.30)	Not applicable
SU-IE	Medium Metro	Large Central Metro	2.21 (1.98-2.46)	Not applicable
SU-IE	Small Metro	Large Central Metro	2.04 (1.77-2.36)	Not applicable
SU-IE	Micropolitan (Nonmetro)	Large Central Metro	2.39 (2.08-2.75)	Not applicable
SU-IE	NonCore (Nonmetro)	Large Central Metro	2.20 (1.86-2.62)	Not applicable

Rate ratios were calculated by dividing the mortality rate in the comparison group by the corresponding rate in the reference group. Confidence intervals were estimated using the delta method. Age-adjusted rate ratios were not calculated for fixed age strata or urban-rural groups. For race-specific comparisons, age-adjusted rates were standardized to the 2000 U.S. standard population. *Age-adjusted rate ratios were not calculated for comparisons between fixed age strata. †The age-adjusted rate ratio could not be estimated because the corresponding CDC WONDER age-adjusted rate rounded to zero owing to sparse events. ‡Estimates were unavailable because CDC WONDER suppressed the underlying data. **Abbreviations:** CI, confidence interval; IE, infective endocarditis; NA, not applicable; RR, rate ratio; SU-IE, substance use–associated infective endocarditis.
